# Is video creation more effective than self-exercise in motor skill learning?

**DOI:** 10.3389/fpsyg.2022.1032680

**Published:** 2022-10-18

**Authors:** Qiudong Xia, Lu’an Ke, Zheng Zheng

**Affiliations:** ^1^Department of Physical Education, Zhejiang Gongshang University, Hangzhou, China; ^2^Ministry of Public Foundation, Zhejiang College of Shanghai University of Finance and Economics, Jinhua, China

**Keywords:** video creation, self-exercise, motor skill learning, intrinsic motivation, video learning

## Abstract

Parallel to the tremendous growth and expansion of video technology, it is easy and enjoyable for students to create a video as a learning activity. However, most previous studies primarily focused on declarative knowledge learning (e.g., language learning, science learning) rather than motor skill learning. The current study aimed to investigate whether creating and sharing a video with classmates would be more effective than merely creating a video and self-exercise to learn a motor skill in terms of intrinsic motivation, perseverance in learning, learning satisfaction, and roller-skating skill. Partially consistent with our hypothesis, we found that creating and sharing a video with classmates increased students’ intrinsic motivation, perseverance in motor tasks, and learning satisfaction, but not roller-skating skill, followed by merely creating a video and then self-exercise. The findings have an important implication for motor skills learning: during teaching motor skills, teachers can use encourage students to create and share a video with classmates as a homework activity to increase students’ intrinsic motivation, perseverance in motor tasks, and learning satisfaction.

## Introduction

Technology has developed significantly, and its impacts are inevitable on education in multiple disciplines (e.g., language learning, science learning, motor skills; Aguilar and Pifarre Turmo, 2019; Chang et al., 2020; Zainuddin and Che’ Lah, 2022). As the nature of dynamic and multichannel presenting learning content, learning from a video is immensely popular and an essential ingredient of many contemporary instructional approaches, such as massive open online courses, flipped classrooms, and even traditional classrooms (Noetel et al., 2021; Rekik et al., 2021). However, previous studies primarily consider videos to deliver learning content to students (Pi et al., 2021, 2022).

Parallel to the tremendous growth and expansion of video technology, it is easy and enjoyable for students to create a video as a learning activity (Greene and Crespi, 2012; Hoogerheide et al., 2019). Some emerging studies are interested in the effects of creating videos by students (Sari et al., 2020; Zainuddin and Che’ Lah, 2022). Creating a video offers students a chance to exercise and self-assessment. During creating a video, students can develop their interpretation, capture, review, and evaluate their performance on their own or by seeking their peers’ views (Huang, 2015; Zainuddin and Che’ Lah, 2022).

Recent research on learning strategies has shown that creating a video can be an effective strategy for learning (Hoogerheide et al., 2019; Erlangga, 2021; Zainuddin and Che’ Lah, 2022). For example, the study by Zainuddin and Che’ Lah (2022) tested the effects of video creation in English classrooms. They found that video creation increased students’ confidence and eventually improved their speaking skills. Similar results were obtained by Hoogerheide et al. (2019) with different learning content (i.e., biology). Again, students creating a video consistently experienced more enjoyment, which may stimulate engagement factors such as perseverance in learning, and showed higher learning satisfaction and better learning performance compared to restudy (Garland, 1982). Perseverance in learning refers to persistence at a learning task, which is evidenced to can be promoted by task enjoyment (Leonard and Weitz, 1971). The benefits of video creation have since been replicated in other studies (Huang, 2015; Sari et al., 2020; Erlangga, 2021; Jung, 2021).

However, most previous studies primarily focused on declarative knowledge learning (e.g., language learning, science learning) rather than motor skill learning. There are some differences between declarative knowledge and motor skill learning (Anderson, 1982). Specifically, declarative knowledge is ‘know-what’ knowledge (e.g., facts, concepts, and theory); motor skill is ‘know-how’ knowledge (e.g., roller-skating, playing ping-pong, and swimming). Therefore, motor skill learning includes learning declarative knowledge and mastering how to enact the skill *via* exercise (van Abswoude et al., 2019). Physical educators struggle with the challenges of creating opportunities for all students to engage in exercise and self-assessment daily, such as roller-skating (Finkenberg et al., 2005). Creating a video as a learning activity can address these challenges by encouraging students to show motor skills, develop their interpretation, capture, review, and evaluate their performance on their own (Huang, 2015; Zainuddin and Che’ Lah, 2022).

Researchers have proposed several potential mechanisms for the effects of creating video. First, the exercise effect postulates that exercise has beneficiary effects on motor skill consolidation (Chen et al., 2019; Khan et al., 2022). Exercise helps students acquire motor memory, which is stored in the form of cortical spine plasticity (Yang et al., 2009; Hayashi-Takagi et al., 2015). During creating a video to show the learned motor skill, students engage in exercise that is effective for motor skill learning (Chen et al., 2019; Khan et al., 2022).

Second, the self-assessment effect builds on empirical studies and suggests that creating a video enables students to self-assess related to their learning and performance by viewing a created video (Paul et al., 1998; Huang, 2015; Ritchie, 2016; Zainuddin and Che’ Lah, 2022). Consequently, self-assessment enhances their self-reflection and adjustment. Some studies have shown that creating video raise students’ awareness of self-assessment by viewing a created video and improves learning performance in the oral presentation (Ritchie, 2016; Tailab and Marsh, 2020).

Third, the social facilitation effect in motor tasks argues that the mere presence of others increases students’ arousal level, intrinsic motivation, and elicits the perception of evaluation by others, and thus influences their performance in motor tasks (Cottrell, 1968; Strauss, 2002). Intrinsic motivation is an essential component of learning, which refers to motivation that originates from within students and consists of spontaneous interest and mastery (Ryan and Deci, 2000). It is a common phenomenon for students to create videos and share them with their classmates later (Huang, 2015, 2021). Therefore, students are aware of the potential audience (i.e., peers) while creating videos. Research has shown that merely believing that someone else is watching you (i.e., a fictitious audience) can evoke arousal (Somerville et al., 2013; Hoogerheide et al., 2018). Based on empirical studies on the social facilitation hypothesis in motor tasks, the mere presence of others facilitates motor skills that have been formulated (Zajonc, 1965; Strauss, 2002).

Although accumulative studies confirmed the benefits of creating a video on students’ intrinsic motivation, engagement, and learning performance (Huang, 2015; Hoogerheide et al., 2018; Erlangga, 2021), it is yet unclear whether creating a video is an effective strategy in motor skills and which working mechanism is responsible for such effects. Specifically, it is unclear whether the benefits of creating a video result from engaging in exercise, self-assessment, or others presence.

The present study tested whether creating and sharing a video with classmates or not would improve students’ motor skill learning (i.e., roller-skating) compared to the self-exercise condition. To control time on task, students created videos or did self-exercise in a physical class. Merely creating a video and self-exercise were used as control conditions, which also encourages exercise and self-assessment by viewing the created video but lacks others presence. Furthermore, compared to creating and sharing a video with classmates, self-exercise lacks self-assessment and others presence. We examined effects on intrinsic motivation, perseverance in motor tasks, learning satisfaction, and roller-skating skill. Based on previous studies and theories, we proposed the following hypotheses.

*H*1: Students who create and share a video with classmates will show higher intrinsic motivation, followed by those who merely create a video and, finally, those who do self-exercise.

*H*2: Students who create and share a video with classmates will show higher perseverance in motor tasks, followed by those who merely create a video and, finally, those who do self-exercise.

*H*3: Students who create and share a video with classmates will show better motor skills, followed by those who merely create a video and, finally, those who do self-exercise.

## Materials and methods

### Participants and design

A total of 160 students (79 females and 81 males, mean age = 20.08, *SD* = 1.28, Range: 18–22 years) from six classes in one Chinese university participated in the study. None of their majors are physics or physics related. We adopted a between-subjects design and thus randomly assigned two classes of students one of three conditions to learn roller-skating. In the creating and sharing a video with classmates condition, there were 53 students (27 females and 26 males, mean age = 20.04 years, *SD* = 1.26, range: 18–22); In the merely creating a video condition, there were 53 students (27 females and 26 males, mean age = 20.32 years, *SD* = 1.33, range: 19–22); In the self-exercise condition, there were 54 students (25 females and 29 males, mean age = 19.87 years, *SD* = 1.23, range: 18–22). All the students were informed of the learning tasks and volunteered to participate in the study.

### Martials

#### Physical fitness tests

Physical fitness tests included squatting against the wall, standing on one leg with eyes closed, sitting and bending forward, 50 m running, standing long jump, and sit-ups. Each test is related to roller skating learning. Specifically, squatting against the wall tests the basic strength of the legs; standing on one leg with eyes closed tests the balance, sitting and bending forward tests the flexibility of the body; 50 m running tests the displacement speed of the body; standing long jump tests the lower limb explosive force; and sit-ups test the waist strength and core control ability. These tests are from the National Student Physical Health Standards in China (Zhang, 2019).

#### Intrinsic motivation scale

The intrinsic motivation scale was developed by Lin et al. (2020). The scale contains 12 items and includes four dimensions: interest (e.g., “I find this learning activity very interesting”), competence (e.g., “I think I am good at this learning activity”), pressure (e.g., “I’m not nervous at all when doing this study activity”), and value (e.g., “I think this class is very useful to me”). Participants indicated their endorsement of each item on a 7-point scale (e.g., 1 = strongly disagree, 7 = strongly agree). High scores indicated high levels of learning motivation. The Cronbach’s alpha coefficients for the four dimensions in the current study were.54, 0.94, 0.94, and.74, respectively.

#### Perseverance in motor tasks questionnaire

The perseverance in motor tasks questionnaire was adapted from Zhou (1991). The questionnaire measures the degree of students’ persistence when encountering learning difficulties, learning disabilities, or external stimuli in motor skills learning. It includes 14 items, such as “When you find a problem that you could not solve in the past during practice, you will try to solve it again within two or 3 days.” The participants answered each item based on their actual situation using a five-point Likert scale (1 = completely inconsistent; 5 = completely consistent). The higher the score, the higher the persistence of the participants. The Cronbach’s alpha coefficient of the questionnaire in the current study was 0.91.

#### Learning satisfaction scale

The learning satisfaction scale was adapted from Wang (2014). The scale contains 17 items and four dimensions: Teaching, course content, teacher-student interaction, and learning environment and equipment. Teaching contains six items, such as “Teachers’ professional knowledge is very rich, and I have learned new knowledge and skills”; course content contains five items, such as “The task in learning is challenging”; teacher-student interaction contains three items, such as “Through mutual learning, I can fully participate in the learning process”; learning environment and equipment contains three items, such as “Satisfied with the normal operation of teaching.” The students needed to evaluate every item according to their feelings using a five-point Likert scale (1 = completely disagree; 5 = completely agree). The higher the score, the more satisfied students are with teaching. The Cronbach’s alpha coefficients for the four dimensions in the current study were 0.95, 0.96, 0.97, and 0.98, respectively.

#### Roller-skating test

**Figure 1:**
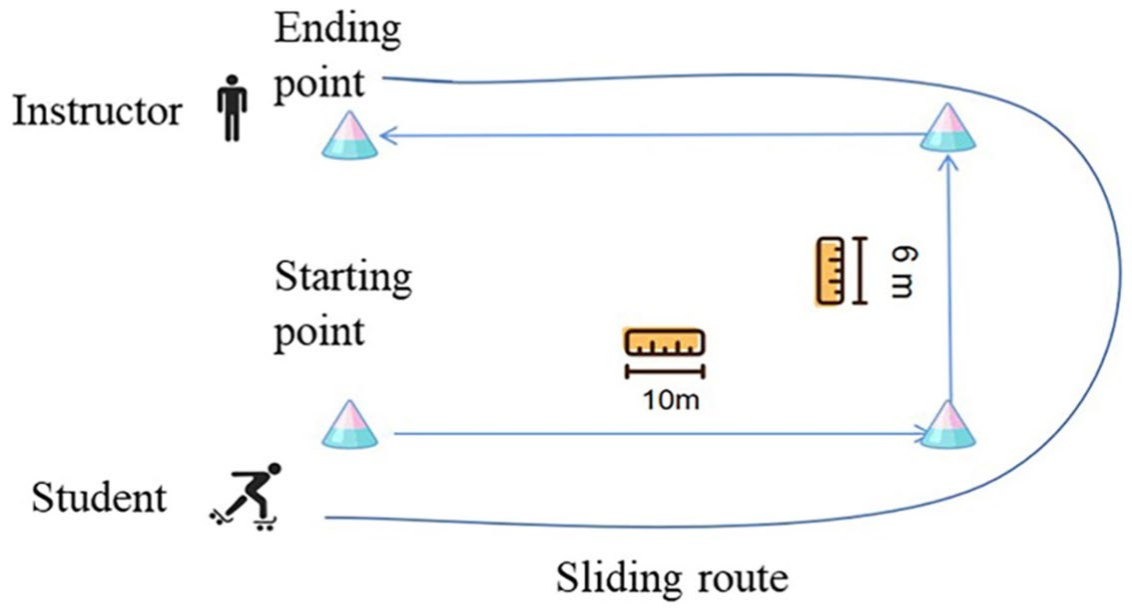
Sliding route of the roller-skating test.

Students completed a total of 26 m roller skating in a roller-skating test. The test included three parts. In part one, students slid forward 10 m from the starting point. In part two, students turned left and slid forward for 6 m. In part three, students turned left and slid forward for 10 m to get the ending point. An instructor at the ending point recorded time. The finish time of 26 m was the roller-skating achievement for students. Shorter the finish time, the better the roller-skating achievement. The test map can be seen in Figure 1.

### Procedure

**Figure 2:**
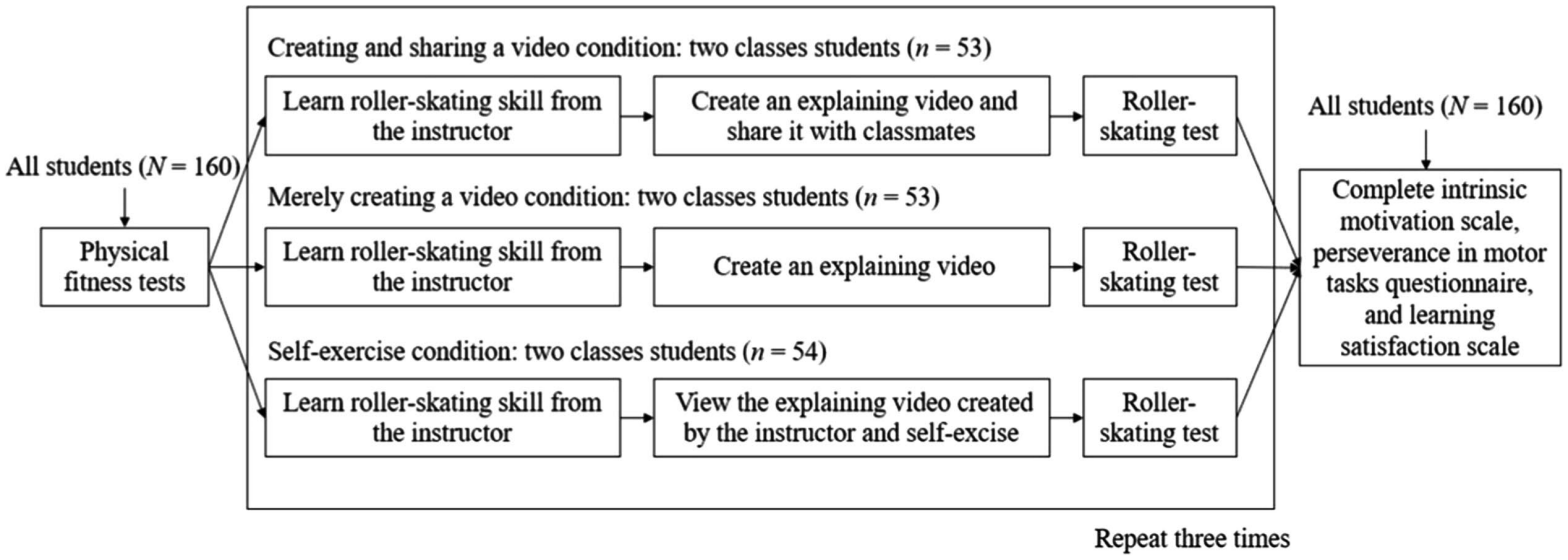
The procedure of the current study.

Figure 2 shows the procedure. First, participants completed physical fitness tests. After that, each class was randomly assigned to one experimental condition, and a male instructor taught the exact content of roller-skating, including standing and sliding. Participants did exercises following the instructor. Then, in the creating and sharing a video condition, the instructor asked the students to create an explaining video in a physical class and share it with their classmates in the WeChat group (i.e., a popular social media in China). In the merely creating a video condition, the instructor asked the students to create an explaining video in a physical class. In the self-exercise condition, students watched the explaining video created by the instructor rather than creating their videos and did exercises themselves in a physical class. At last, all students completed the roller-skating test. These steps were repeated three times. Finally, after the third class, all students finished the questionnaires.

### Data analysis

We first tested whether there were differences in the physical fitness tests among the three conditions. We conducted between-group comparisons to test H1, H2 and H3: intrinsic motivation, perseverance in motor tasks, and performance on the roller-skating test. If there were differences in the physical fitness tests among the three conditions, to eliminate the potential influence of the participants’ physical fitness on the results, we conducted these comparisons with the analysis of covariance (ANCOVA), in which we used the scores on the physical fitness tests as covariates in our models. All analyses were carried out on SPSS 26.

## Result

Table 1 shows the means and standard deviations (*SD*) of all variables in the current study.

**Table 1:** Means and standard deviations (*SD*) of all variables.

	Creating and sharing a video		Merely creating a video		Self-exercise	
	Mean	*SD*	Mean	*SD*	Mean	*SD*
*Physical fitness tests*						
Squatting against the wall (s)	87.70	45.85	108.02	58.80	121.08	55.16
Standing on one leg with eyes closed (s)	65.08	55.96	81.02	63.87	100.48	66.04
Sitting and bending forward (cm)	17.41	6.27	14.60	6.65	16.31	7.02
50 m running (s)	8.51	0.95	8.14	0.93	7.89	1.08
Standing long jump (cm)	186.04	32.98	200.85	33.03	208.67	42.14
Sit-ups	45.70	9.11	41.43	9.30	39.83	9.68
*Intrinsic motivation*						
Interest	5.20	1.17	5.01	1.02	5.12	1.37
Competence	5.37	1.54	5.04	1.35	4.98	1.55
Pressure	5.06	1.33	4.86	1.21	5.04	1.57
Value	5.86	1.26	5.34	1.09	5.38	1.31
Perseverance in motor tasks	4.07	0.48	3.59	0.68	3.69	0.79
*Learning satisfaction*						
Teaching	4.40	0.81	4.17	0.79	3.98	0.92
Classroom content	4.35	0.82	4.07	0.78	3.96	0.95
Teacher-student interaction	4.43	0.80	4.07	0.80	4.04	1.00
Learning environment and equipment	4.40	0.92	4.06	0.86	4.01	1.01
Roller skating tests (s)	23.29	14.16	20.50	10.60	20.85	9.30

### Physical fitness tests

We first tested the differences in physical fitness tests among the three conditions. The results of one-way ANOVAs showed significant differences in squatting against the wall, *F*(2,157) = 5.27, *p* = 0.006, *η_p_*^2^ = 0.06. *Post hoc* comparison (*LSD*) showed that participants’ time in the creating and sharing a video condition was significantly shorter than those in the self-exercise condition (*MD* = −33.38, *p* = 0.002, 95% CI [−53.83, −12.92]).

There were significant differences in standing on one leg with eyes closed, *F*(2,157) = 4.36, *p* = 0.014, *η_p_*^2^ = 0.05. *Post hoc* comparison showed that participants’ time in the creating and sharing a video condition was significantly shorter than those in the self-exercise condition (*MD* = −35.41, *p* = 0.004, 95% CI [−59.13, −11.68]).

There were marginally significant differences in sitting and bending forward, *F*(2,157) = 2.41, *p* = 0.094, *η_p_*^2^ = 0.03. *Post hoc* comparison showed that participants’ distance in the creating and sharing a video condition was significantly longer than those in the merely creating a video condition (*MD* = 2.813, *p* = 0.032, 95% CI [0.26, 5.37]);

There were significant differences in 50 m running, *F*(2,157) = 5.39, *p* = 0.005, *η_p_*^2^ = 0.06. *Post hoc* comparison showed that participants’ time in the creating and sharing a video condition was significantly longer than those in the self-exercise condition (*MD* = 0.62, *p* = 0.001, 95% CI [0.25, 1.00]).

There were significant differences in standing long jump, *F*(2,157) = 5.34, *p* = 0.006, *η_p_*^2^ = 0.06. *Post hoc* comparison showed that participants’ distance in the creating and sharing a video condition was significantly shorter than those in the merely creating a video condition (*MD* = −14.81, *p* = 0.038, 95% CI [−28.75, −0.86]), and was significantly shorter than those in the self-exercise condition (*MD* = −22.64, *p* = 0.002, 95% CI [−36.52, −8.75]);

There were significant differences in sit-ups, *F*(2,157) = 5.59, *p* = 0.004, *η_p_*^2^ = 0.07, and *post hoc* comparison showed that participants’ number in the creating and sharing a video condition was significantly more than those in the merely creating a video condition (*MD* = 4.26, *p* = 0.020, 95% CI [0.67, 7.86]), and was significantly more than those in the self-exercise condition (*MD* = 5.87, *p* = 0.001, 95% CI [2.29, 9.44]).

In sum, the above results indicated that participants in the three conditions were different in physical fitness. Therefore, we conducted ANCOVAs to test the effects of creating video on motor skill learning, the scores on the physical fitness tests as covariates.

### Intrinsic motivation

For the interest, competence, and pressure dimensions, there was no significant difference between the three conditions, *F*(2,151) = 0.59, *p* = 0.556, *η_p_*^2^ = 0.01; *F*(2,151) = 0.63, *p* = 0.536, *η_p_*^2^ = 0.01; *F* (2,151) = 0.50, *p* = 0.607, *η_p_*^2^ = 0.01.

**Figure 3:**
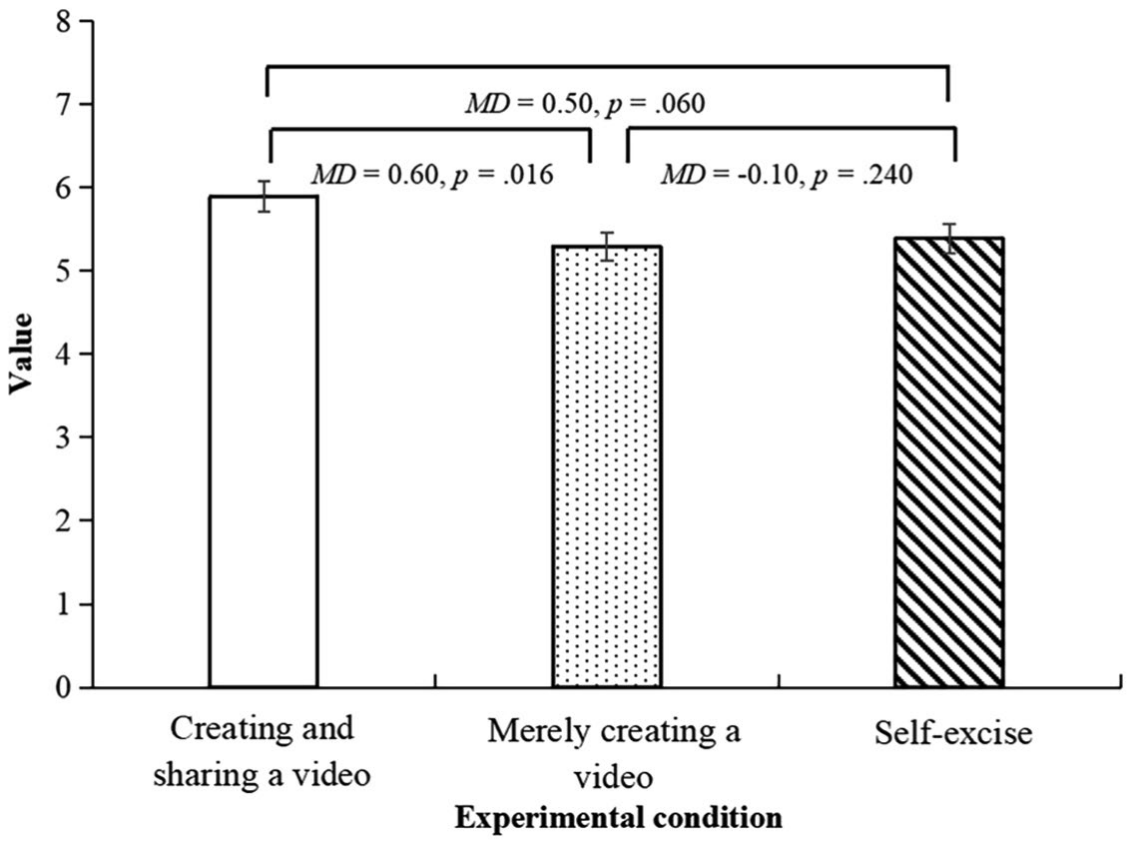
The bar chart for the value dimension.

For the value dimension, there were significant differences between the three conditions, *F*(2,151) = 3.15, *p* = 0.046, *η_p_*^2^ = 0.04. The *post hoc* tests showed that participants in the creating and sharing a video condition reported a significantly higher score than those in the merely creating a video condition (*MD* = 0.60, *p* = 0.016, 95% CI [0.11, 1.10]) and a higher score, in a marginally significant level, than those in the self-exercise condition (*MD* = 0.50, *p* = 0.060, 95% CI [−0.02, 1.03]). See Figure 3.

### Perseverance in learning

**Figure 4:**
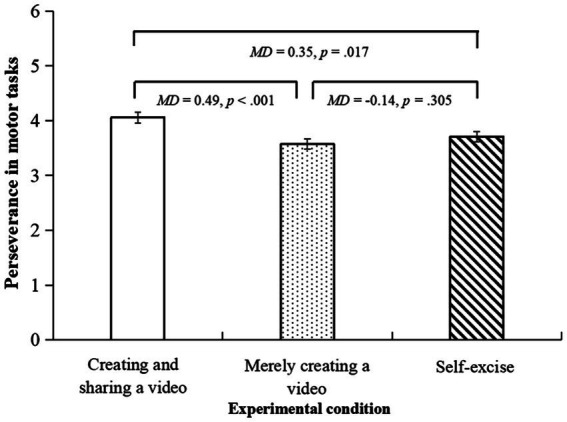
The bar chart for perseverance in motor tasks.

The ANCOVA showed that there were significant differences in the score of the perseverance in motor tasks questionnaire between the three conditions, *F*(2,151) = 6.42, *p* = 0.002, *η_p_*^2^ = 0.08. The *post hoc* tests showed that participants in the creating and sharing a video condition reported a significantly higher score than those in the merely creating a video condition (*MD* = 0.49, *p* < 0.001, 95% CI [0.22, 0.76]) and the self-exercise condition (*MD* = 0.35, *p* = 0.017, 95% CI [0.06, 0.64]). See Figure 4.

### Learning satisfaction

**Figure 5:**
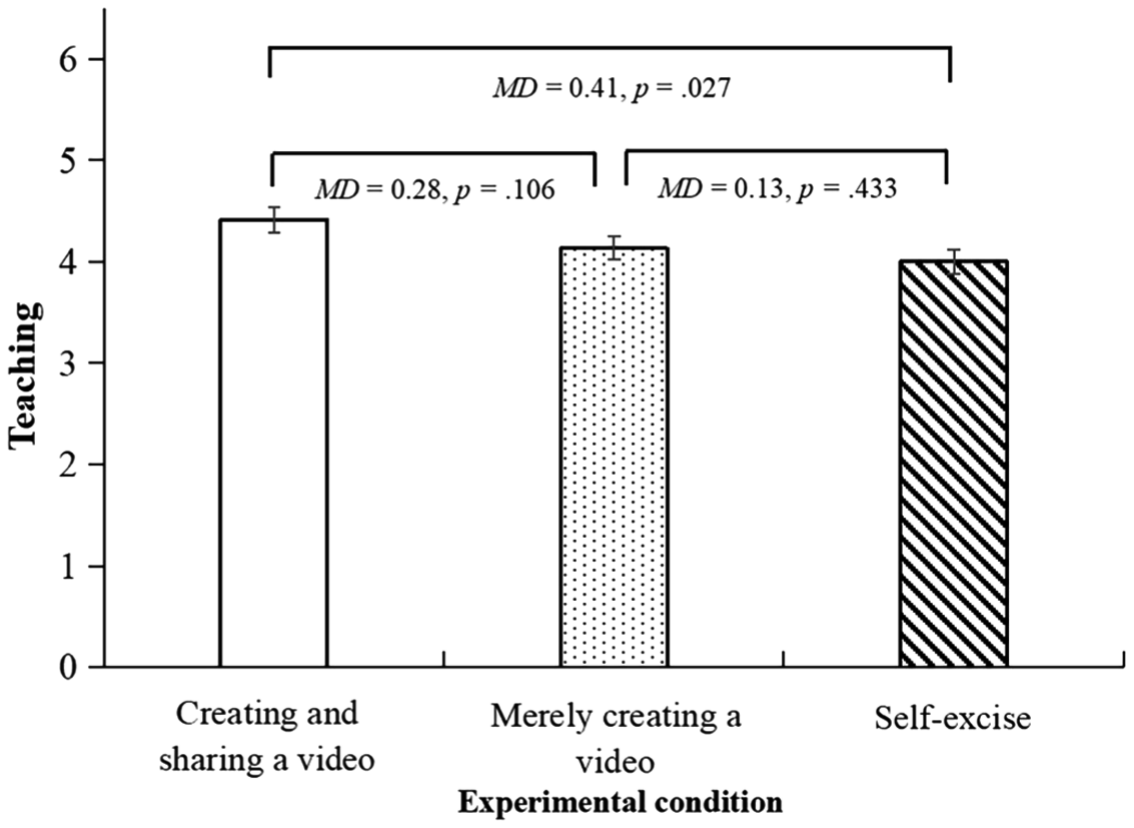
The bar chart for the teaching dimension.

For the teaching dimension, there were differences in a marginally significant level between the three conditions, *F*(2,151) = 2.60, *p* = 0.078, *η_p_^2^* = 0.03. The *post hoc* test showed that participants in the creating and sharing a video condition reported a significantly higher score than those in the self-exercise condition (*MD* = 0.41, *p* = 0.027, 95% CI [0.05, 0.77]). See Figure 5.

**Figure 6:**
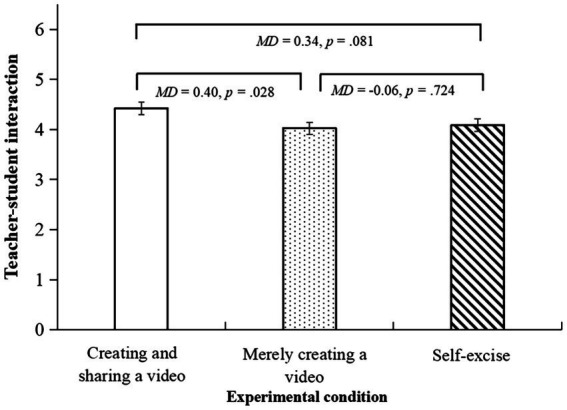
The bar chart for the teacher-student interaction dimension.

For the teacher-student interaction dimension, there were a difference in a marginally significant level between the three conditions, *F* (2,151) = 2.65, *p* = 0.074, *η_p_*^2^ = 0.03. The *post hoc* test showed that participants in the creating and sharing a video condition reported a significantly higher score than those in the merely creating a video condition (*MD* = 0.40, *p* = 0.028, 95% CI [0.04, 0.75]), and a higher score, in a marginally significant level, than those in the self-exercise condition (*MD* = 0.34, *p* = 0.08, 95% CI [−0.04, 0.71]). See Figure 6.

For the course content and the learning environment and equipment dimensions, there was no significant difference between the three conditions, *F*(2,151) = 2.23, *p* = 0.111, *η_p_*^2^ = 0.03; *F*(2,151) = 1.95, *p* = 0.146, *η_p_*^2^ = 0.03.

### Roller-skating test

We calculated the average time of three roller-skating tests as the final scores. The result showed no significant difference between the three conditions, *F*(2,151) = 0.68, *p* = 0.508, *η_p_*^2^ = 0.01.

## Discussion

The current study aimed to investigate whether creating and sharing a video with classmates would be more effective than merely creating a video and self-exercise to learn a motor skill in terms of intrinsic motivation, perseverance in learning, learning satisfaction, and roller-skating skill. Partially consistent with our hypothesis, we found that creating and sharing a video with classmates increased students’ intrinsic motivation, perseverance in motor tasks, and learning satisfaction, but not roller-skating skill, followed by merely creating a video and then self-exercise. The results broadened our understanding of the effects of creating a video on motor skills learning.

In line with our first and second hypotheses that creating a video improved intrinsic motivation and perseverance in motor tasks compared with self-exercise, we confirmed the benefits of creating a video on students’ intrinsic motivation (i.e., the value dimension) and perseverance in motor tasks compared to self-exercise. The results were consistent with previous studies, showing creating a video increases students’ intrinsic motivation (i.e., value) and learning satisfaction (Greene and Crespi, 2012; Yang and Wu, 2012; Huang, 2015; Zainuddin and Che’ Lah, 2022). Previous studies have shown that creating a video encourages students’ engagement and supports support authentic learning (Kearney and Schuck, 2003; Schuck and Kearney, 2004; Greene and Crespi, 2012). Some researchers defined behavioral engagement as effort and perseverance in learning (Lee, 2014). Furthermore, intrinsic motivation is positively associated with enhanced perseverance in learning (Larson and Rusk, 2011). Students may have been more inclined to persevere with the difficult material due to intrinsic motivation (Logan et al., 2011).

In contrast to our third hypothesis that creating and sharing a video with classmates facilitated roller-skating skills most, we did not observe differences in the roller-skating skills among the three conditions. The results suggested that self-exercise was enough to lead to good performance in the roller-skating test. Hoogerheide et al. (2019) found that creating videos did not outperform the summarizing condition, like the self-exercise condition in the present study. Additionally, the study by Sari et al. (2020) also did not observe students’ English skills/proficiency was improved by creating video sharing on YouTube. Therefore, exercise, self-assessment, or other presence seem to not contribute to the mechanisms underlying the benefits of creating video. In contrast, teaching expectancy might play an essential role in the benefits of creating videos observed in previous studies (Hoogerheide et al., 2018, 2019). The studies on teaching by creating videos have shown that students in the teaching on video condition consistently outperform and report more engagement (Hoogerheide et al., 2016, 2018, 2019; van Brussel et al., 2021). Future work should test whether a teaching expectancy enhances the benefits of creating a video on learning motor skills.

This study has the following limitations. First, this study did not test the long-term effect of creating and sharing a video with classmates. We observed that creating and sharing a video increased students’ intrinsic motivation and perseverance in motor tasks. Intrinsic motivation is a powerful “engine” of learning (Larson and Rusk, 2011). Intrinsic motivation is associated with enhanced engagement and perseverance in learning but with greater use of meta-cognitive strategies and deeper information processing (Larson and Rusk, 2011). Therefore, the benefits of creating and sharing a video with classmates might be exhibited over time. Future studies should test the long-term effect of creating and sharing a video with classmates. Second, we did not analyze the videos created by students. Previous studies on generative learning have shown that the quantity and quality of generative learning activities mediate the effect of generative learning strategies (e.g., self-explaining) on learning performance (Baars et al., 2018; Hefter, 2021; Bichler et al., 2022). Future research should further investigate the mediating role of the quantity and quality of the videos created by students.

Although there were some limitations, the present study advances our understanding of the various effects of two types of creating video compared to self-exercise. With the rapid development of video technology, it is easy for students to create a video as an enjoyable activity (Greene and Crespi, 2012; Hoogerheide et al., 2019). The present study tested the various effects of video creation by including three conditions: creating and sharing a video with classmates, merely creating a video, and self-exercise. Additionally, we measured students’ subjective perceptions and their objective performance on the roller-skating test. Our results suggest that video creation enhances students’ intrinsic motivation, perseverance in motor tasks, and learning satisfaction compared to self-exercise; sharing with classmates can further enhance their intrinsic motivation, perseverance in motor tasks, and learning satisfaction; self-exercise guarantees good performance on motor skill.

In conclusion, the main finding of the present study was that students who created and shared a video with classmates reported higher intrinsic motivation, perseverance in motor tasks, and learning satisfaction, but not the roller-skating skill, followed by those who merely creating a video and then those who self-exercise. With the easy availability of video production software, the findings have an important implication for motor skills learning: during teaching motor skills, teachers can use encourage students to create and share a video with classmates as a homework activity to increase students’ intrinsic motivation, perseverance in motor tasks, and learning satisfaction.

## Data availability statement

The original contributions presented in the study are included in the article/supplementary material, further inquiries can be directed to the corresponding author.

## Ethics statement

The studies involving human participants were reviewed and approved by the Ethics Committee of Zhejiang Gongshang University. The participants provided their written informed consent to participate in this study.

## Author contributions

QX: conceptualization, methodology, writing – original draft, and writing-reviewing and editing. LK: investigation, data curation, visualization, and methodology. ZZ: supervision and conceptualization. All authors contributed to the article and approved the submitted version.

## Conflict of interest

The authors declare that the research was conducted in the absence of any commercial or financial relationships that could be construed as a potential conflict of interest.

## Publisher’s note

All claims expressed in this article are solely those of the authors and do not necessarily represent those of their affiliated organizations, or those of the publisher, the editors and the reviewers. Any product that may be evaluated in this article, or claim that may be made by its manufacturer, is not guaranteed or endorsed by the publisher.
