# Corrigendum: Is video creation more effective than self-exercise in motor skill learning?

**DOI:** 10.3389/fpsyg.2024.1359917

**Published:** 2024-02-02

**Authors:** Qiudong Xia, Lu'an Ke, Zheng Zheng

**Affiliations:** ^1^Department of Physical Education, Zhejiang Gongshang University, Hangzhou, China; ^2^Ministry of Public Foundation, Zhejiang College of Shanghai University of Finance and Economics, Jinhua, China

**Keywords:** video creation, self-exercise, motor skill learning, intrinsic motivation, video learning

In the published article, there was an error in the affiliations for author Zheng Zheng. Instead of affiliations 2, “College of Business, Shanghai University of Finance and Economics, Shanghai, China”, and 3, “Physical Education Teaching and Research Office of Public Foundation Department, Zhejiang University of Finance and Economics, Hangzhou, Zhejiang, China”, it should just be “2 Ministry of Public Foundation, Zhejiang College of Shanghai University of Finance and Economics, Jinhua, China.”

The authors apologize for this error and state that this does not change the scientific conclusions of the article in any way. The original article has been updated.

